# Empirical Bayesian selection of hypothesis testing procedures for analysis of digital gene expression data

**DOI:** 10.1186/1471-2105-14-S17-A21

**Published:** 2013-10-22

**Authors:** Stan Pounds, Cuilan Gao

**Affiliations:** 1 Department of Biostatistics, St. Jude Children’s Research Hospital, Memphis, TN 38105, USA; 2 Department of Mathematics, University of Tennessee Chattanooga, Chattanooga, TN 37403, USA

## Background

Differential expression analysis of digital gene expression data involves performing a large number of hypothesis tests that compare the expression count data of each gene or transcript across two or more biological conditions. The assumptions of any specific hypothesis-testing method will probably not be valid for each of a very large number of genes. Thus, computational evaluation of assumptions should be incorporated into the analysis to select an appropriate hypothesis-testing method for each gene.

## Materials and methods

Here, we generalize earlier work to introduce two novel procedures that use estimates of the empirical Bayesian probability (EBP) of overdispersion to select or combine results of a standard Poisson likelihood ratio test and a quasi-likelihood test for each gene. These EBP-based procedures simultaneously evaluate the Poisson-distribution assumption and account for multiple testing. With adequate power to detect overdispersion, the new procedures select the standard likelihood test for each gene with Poisson-distributed counts and the quasi-likelihood test for each gene with overdispersed counts.

## Results

The new procedures outperformed previously published methods in many simulation studies. We also present a real-data analysis example and discuss how the framework used to develop the new procedures may be generalized to further enhance performance.

